# Efficient Model of the Interaction of Elastomeric Filler with an Open Shell and a Chrome-Plated Shaft in a Dry Friction Damper

**DOI:** 10.3390/ma15134671

**Published:** 2022-07-03

**Authors:** Maciej Dutkiewicz, Andrii Velychkovych, Ivan Shatskyi, Vasyl Shopa

**Affiliations:** 1 Faculty of Civil and Environmental Engineering and Architecture, Bydgoszcz University of Science and Technology, Kaliskiego 7, 85-796 Bydgoszcz, Poland; 2 Department of Construction and Civil Engineering, Ivano-Frankivsk National Technical University of Oil and Gas, Karpatska Str. 15, 76019 Ivano-Frankivsk, Ukraine; a_velychkovych@ukr.net; 3 Laboratory of Modeling of Damping Systems, Pidstryhach-Institute for Applied Problems in Mechanics and Mathematics of the National Academy of Sciences of Ukraine, Mykytynetska Str. 3, 76002 Ivano-Frankivsk, Ukraine; ipshatsky@gmail.com (I.S.); vasyl.shopa@gmail.com (V.S.)

**Keywords:** elastomeric filler, open shell, chrome-plated shaft, damping, coefficient of friction

## Abstract

The results of a study of the contact interaction of an open shell and a chrome-plated shaft with elastomeric filler installed coaxially are presented. The considered contact system is a model of the original design of the shell damper of dry friction. The design feature is the following: the bearing link of the damper is a thin-walled cylindrical shell with a cut along the generatrix; the working body of the damper is elastomeric filler; a hollow chrome-plated shaft centers the damper elements and allows it to be used in technological processes with the presence of aggressive and abrasive-containing media. The mechanical-mathematical modeling of the behavior of the presented damper under the conditions of operational loads has been carried out. The idea of identifying the properties of a cut isotropic shell, which bends under the conditions of a nonaxisymmetric contact load, and a strongly orthotropic continuous shell is applied. As a result, dependences were obtained to determine the rigidity and the maximum allowable load of the damper. The effect of the coefficient of friction of the contact pairs elastomer-shell and elastomer-shaft on the damper performance properties has been studied. A technique for the quasi-static analysis of structural damping in non-mobile, non-conservative shell systems with deforming filler has been developed. The hysteresis loops of the damper under a nonmonotonic load are constructed, the dependence of the amount of dissipated energy on the cycle asymmetry coefficient is found. An analysis of the results obtained showed that the use of open shells in friction shock absorbers can significantly reduce their rigidity compared to solid shells and thereby reduce the resonant frequencies of the dynamic system. This circumstance makes such vibration isolators particularly attractive for use in superresonance vibrators as working modules of drilling shock absorbers and elastic hangers of sucker rods in oil and gas production.

## 1. Introduction

One of the main directions of the development of modern mechanical engineering and construction is a constant increase in the resource of structures while reducing material consumption and improving performance. The presence of such mutually competing trends requires the improvement of design methods and the maintenance of high reliability parameters in the operation of products.

The operation of modern machines, devices, and structures is accompanied by the appearance of vibrational actions. In most cases, vibrations reduce the strength, reliability, and durability of structural elements and have a detrimental effect on the health of operating personnel. Thus, the task of the high-quality damping of vibration is relevant both in technical and social terms.

Friction devices have long been used in engineering and construction to absorb and dissipate energy. Dry friction dampers are widely used in vibration protection systems due to their simple design, inexpensive maintenance, and relatively small dimensions [1,2].

In particular, dry friction damping technology is an effective means of reducing the vibration load in gas turbine engines [3,4], paddle disks [5,6], and cardan drives of helicopter tail rotors [7]. Friction effects in the so-called Coulomb damper are widely used in material mixing equipment and automatic washing machines [8].

Today, much attention is paid to the modernization and design of building frames with the use of passive dampers to dissipate the energy of external disturbances [9,10,11,12]. In recent years, a number of designs of friction dampers have been developed to improve the seismic characteristics of industrial and civil structures [13]. In the wires of power lines and ground wires, under the influence of wind, stable vibration can occur in the frequency range characteristic of these objects [14]. Aeolian vibration with a large swing and duration of oscillation can lead to fatigue damage to the wire. Protection against aeolian vibration is carried out using vibration dampers [15]. A difficult task of maintaining the system of the vibration damping of wires of power lines is the diagnostics of shock absorbers slippage [16].

Innovative mining technologies involve complex operations, often associated with abnormal dynamic loads of equipment [17,18]. The design features and specific operation of drill strings and sucker rod strings lead to the occurrence of vibration loads of the downhole tool [19,20,21]. The cardinal way to solve the problem of the vibration protection of drilling and oilfield equipment is based on the use of special vibration protection devices: shock guards and elastic spindles of downhole motors [22,23,24], dynamic dampers and specialized dampers [25,26,27], and elastic suspensions of sucker rod strings [28,29]. The main actuating units of such vibration protection systems are elastic elements and dampers.

**Figure 1:**
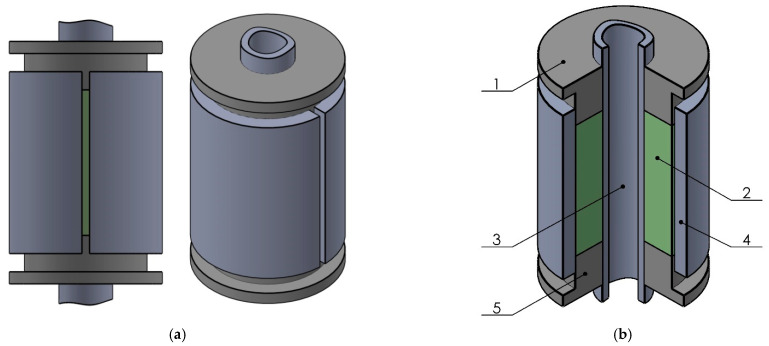
Shell damper of dry friction. (**a**) General view; (**b**) structural scheme: 1—upper piston, 2—elastomeric filler, 3—shaft, 4—cylindrical shell with a cut along the generatrix, 5—lower piston.

For the vibration protection of heavy loaded equipment in the oil and gas, construction, and engineering industries, we suggest using dry friction shell dampers (Figure 1a) [30,31,32]. Vibration protection devices designed on the basis of such dampers are able to operate effectively under high dynamic loads and, at the same time, have small transverse dimensions. The main feature of such structures is the use of an open thin-walled cylindrical shell as the main bearing link. The practical use of shell dampers indicated the need to equip a number of structures with a longitudinal through channel necessary to perform certain technological operations (fixing or centering the device, the passage of the working fluid, cooling the system, etc.). For example, in shell dampers for drilling means of vibration protection, the presence of a through channel is due to the need to pump washing fluid through the drill string for thorough bottom hole cleaning [33,34]. The abrasive environment leads to the rapid wear of steel parts, so hard chromium is applied to the surface of the shaft. For the chromium plating of long shafts—for example, shafts for drilling shock absorbers—an improved technology of chromium plating in a flowing electrolyte is used [35,36]. The use of this technology makes it possible to apply a uniform layer of a protective coating with a low roughness to long parts while ensuring a high coating rate [37].

The general principle of the damper is as follows. The axial load acts on the pistons and forces them to move towards each other, compressing the elastomeric filler (Figure 1b). The filler changes its shape and enters into frictional interaction with the open shell and the shaft. As a result, the shell is deformed within the limits of elasticity and accumulates the potential energy of elastic deformation. When the external overload disappears, the movable parts of the damper return to their original position due to the accumulated energy. Part of the energy of external influences is dissipated, mainly due to mutual slippage with the friction of the filler and the shell with the shaft.

For a directional transformation of the movement of the pistons into the movement of an open shell, the filler used in the damper must easily change its shape (i.e., have a low shear modulus). From this position, the filler material is soft compared to the shell material. On the other hand, in order to cause the shell to deform under contact interaction conditions, the filler material must be weakly compressible (i.e., have a high bulk modulus). These requirements are met to a certain extent by hydroplastics, elastomers, viscous liquids, and granular materials. Currently, the most common fillers for shell dampers are elastomers, the rheological properties of which create an additional reserve for improving the deforming and damping properties of these devices [38,39]. Along with natural rubber, elastomers usually include styrene-butadiene rubber, ethylene-propylene-diene rubber, nitrile rubber, polycaprolactone, butyl rubber, thermoplastic urethane, thermoplastic olefins, ethylene propylene rubber, and products of the special processing of rubber with various additives and inclusions. Works [40,41,42] discuss the processes of the modern production of elastomers and technical rubber, the determination of the physical and mechanical properties of these materials, and typical applications using elastomers.

From the point of view of mechanics, the damper with an open shell and shaft is a deformable system with a dry positional friction. In the mathematical modeling of such systems under the influence of a nonmonotonic load, structurally nonlinear contact problems arise about the frictional interaction of coaxially placed shells with an elastic body.

Contact problems for elastic systems constitute an actual branch of the mechanics of deformable solids [43,44,45,46,47].

Problems on the contact interaction of rod systems with the environment have a wide practical application [48,49,50]. The demand for such studies regularly arises in such areas as the construction and operation of oil and gas wells [51,52,53], the design of pipelines in karst zones [54], areas of abnormal tectonic activity [55,56,57], the driving of construction piles [58,59,60], and the recovery of stuck long tools [61,62,63].

Let us also note the class of problems related to the effect of the contact interaction of the edges of cuts during the bending of thin shells [64,65,66,67,68] described within the model of contact along a line [69,70]. Similar problems for loading by simultaneous strain and bending are solved in the works [71,72,73,74,75]. The elastic and boundary equilibrium of cut thin-walled structures resting on an elastic foundation was studied in [76,77,78,79,80].

A detailed review of publications devoted to the study of dynamical systems with different laws of friction is carried out in articles [81,82,83].

To date, the mechanisms of structural damping or energy dissipation under nonmonotonic conditions—in particular, under the cyclic loading of nominally immobile or slow-moving joints and systems—can be considered sufficiently studied [84,85,86].

For the analytical study of the frictional interaction of deformable solids, approaches using the models and methods of the theories of rods, shells, and plates are effective. Dimension reduction in the mathematical models of elastic bodies simplifies the derivation and obtains solutions of equations in comparison with the three-dimensional problems of elasticity theory. The main difficulties of contact problems for thin-walled structures arise at the stage of modeling the object of study and choosing an adequate theory. Contact problems for thin-walled elements have their own specifics associated with the appearance of irregular contact stresses and additional detachment zones [87,88,89]. In the presence of dry friction on the contact surfaces, problematic issues become even greater.

In their previous work, the authors described the models and methods of the analytical and numerical-analytical study of the frictional interaction of thin solid shells with compliant weakly compressed filler for the action of monotonic and non-monotonous loads [90,91,92,93]. The problems of studying the stressed state of metal–polymer composites are also known [94,95,96,97]. Analytical studies of the contact interaction of elastomeric filler with an open shell have not been carried out.

The purpose of this study is to develop a theory for calculating a shell damper, the principle of which is based on the frictional interaction of an elastomeric filler with a cut cylindrical shell and a shaft. To do this, it is necessary to develop models of the shell damper components that are adequate in terms of rigor and solve the contact problem of the frictional interaction of elastomeric filler with an open shell and a shaft under a nonmonotonic load. Therefore, on the basis of the conducted research, it is necessary to obtain an engineering method for determining the rigidity, strength, and damping properties of a shell damper. The sequence of listed tasks describes the structure of the article. The publication ends with a short discussion and conclusions.

## 2. Materials and Methods

**Figure 2:**
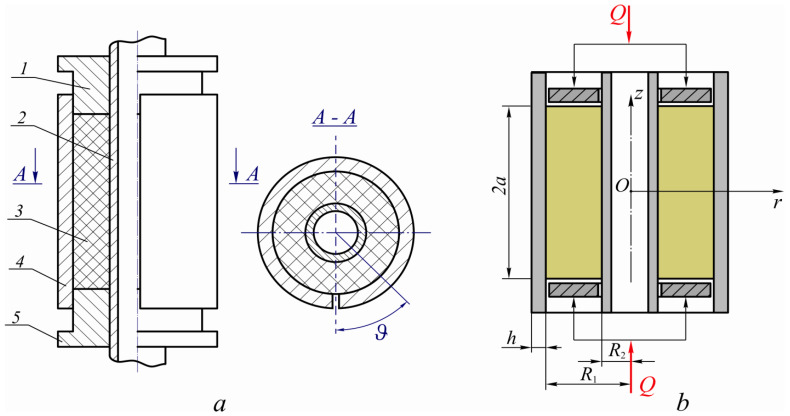
Computational scheme of the damper with an open shell, equipped with a through bore: (**a**) scheme of the interaction of the damper components; 1—upper piston, 2—shaft, 3—elastomeric filler, 4—cylindrical shell with a cut along the generatrix, 5—lower piston; (**b**) scheme of the computational model; 2a—length of the filler, $R_{1}$ and $R_{2}$—radii of outer and inner shells, h—the thickness of the cut shell, Q—external load.

For the analytical modeling of a dry friction damper with an open shell, let us consider the system in Figure 2a, composed of coaxially mounted solid inner shell 2 and outer shell 4 cut along the generatrix. These shells are separated by a deformable filler 3, compressed at the ends by the rigid annular pistons 1 and 5.

Let us introduce a cylindrical coordinate system r,ϑ,z. As a result of the symmetry of the structure, the center of the coordinate system will be placed at the level of the plane equidistant from the ends of the pistons (Figure 2b).

Let the hollow cylinder with the length 2a, an outer radius $R_{1}$, and an inner radius $R_{2}$ fill the space between the coaxially mounted outer open shell with the thickness h and the inner solid shell. An external load Q is transferred to the ends of the cylinder through absolutely rigid smooth pistons. The filler and shells interact with dry friction. It is necessary to investigate the stress-strain state of the system under the action of a given monotonically increasing load. 

The main contribution to the compliance of the system will be made by the opening of the outer shell with a cut along the generatrix. Therefore, to model the elastic element, we will take the following assumptions. We assume that the filler is incompressible, and the inner shell is absolutely rigid. The outer shell with a cut is modeled by a continuous, equivalent, strongly orthotropic, momentless shell. The elasticity and strength characteristics of such an equivalent shell are determined by solving an additional problem of loading a cut shell with internal pressure [98].

In view of the accepted assumptions and using the results of [99,100], we write the equilibrium equations averaged over the cross section and the incompressibility equations for the deformable filler:

(1) $$\frac{d\sigma_{\zeta }}{d\zeta }+2a\frac{R_{1}\tau_{1}-R_{2}\tau_{2}}{R_{1}^{2}-R_{2}^{2}}=0,$$

(2) $$\frac{du}{d\zeta }+2a\frac{R_{1}w_{1}-R_{2}w_{2}}{R_{1}^{2}-R_{2}^{2}}=0.$$

and the expressions for the normal displacements of its cylindrical surfaces from the action of radial and axial stresses

$$\frac{w_{1}}{R_{1}}=\frac{1}{E\left(R_{1}^{2}-R_{2}^{2}\right)}\left\{\left(\frac{3}{2}R_{2}^{2}+\frac{1}{2}R_{1}^{2}\right)\sigma_{1}-2R_{2}^{2}\sigma_{2}\right\}-\frac{1}{2E}\sigma_{\zeta },$$

(3) $$\frac{w_{2}}{R_{2}}=\frac{1}{E\left(R_{1}^{2}-R_{2}^{2}\right)}\left\{2R_{1}^{2}\sigma_{1}-\left(\frac{3}{2}R_{1}^{2}+\frac{1}{2}R_{2}^{2}\right)\sigma_{2}\right\}-\frac{1}{2E}\sigma_{\zeta }.$$

Here, ζ=z/a∈(−1,1) is the dimensionless axial coordinate, $\sigma_{\zeta }$ and u are the axial stress and displacement, $w_{1}$, $w_{2}$ are the radial displacements of the outer and inner side surfaces of the filler, $\sigma_{i},\tau_{i}$ are the normal and tangential contact stresses on the contact surfaces $r=R_{i}(i=1,2)$, and E is the Young’s modulus of the filler material.

For the radial displacement of the outer orthotropic shell, under the assumptions made, we obtain:

(4) $$\frac{w_{1}^{sh}}{R_{1}}=-\frac{\sigma_{1}R_{1}}{E_{e}h},\zeta \in \left(-1,\;1\right),$$

where $E_{e}=\frac{1}{18}\frac{h^{2}}{R_{1}^{2}}E_{0}\;$ is an equivalent modulus of elasticity [98] and $E_{0}$ is a Young’s modulus of the shell material with a cut.

For a rigid shaft,

(5) $$w_{2}^{sh}=0,\;\;\zeta \in \left(-1,1\right).$$

Both the shell and the barrel in the axial direction are considered nondeformable, so their axial displacements are absent:

(6) $$u_{i}^{sh}=0,\;\;i=1,2,\;\;\zeta \in \left(-1,1\right).$$

On the contact surfaces, the filler and shells interact with Coulomb friction:

(7) $$w_{i}=w_{i}^{sh},\;\sigma_{i}\leq 0;$$

(8) $$\tau_{i}={\left(-1\right)}^{i}f_{i}\sigma_{i}\mathrm{sgn}\varsigma ,\;i=1,2,\;\zeta \in \left(-1,1\right),$$

where $f_{i}$ is the coefficient of friction on the contact surfaces $r=R_{i}$.

Since the shaft is assumed to be absolutely rigid and the orthotropic shell is assumed to be nondeformable in the direction z, the slippage area covers the entire contact area. The sign of the contact stresses corresponds to the active load phase with increasing force Q.

Satisfying the kinematic conditions of contact (7), with the help of Equations (3)–(5), we obtain the relation:

$$\frac{1}{E\left(R_{1}^{2}-R_{2}^{2}\right)}\left\{\left(\frac{3}{2}R_{2}^{2}+\frac{1}{2}R_{1}^{2}\right)\sigma_{1}-2R_{2}^{2}\sigma_{2}\right\}-\frac{1}{2E}\sigma_{\zeta }=-\frac{R_{1}^{2}}{{\bar{E}}_{e}h}\sigma_{1}\;,\;$$

(9) $$\frac{1}{E\left(R_{1}^{2}-R_{2}^{2}\right)}\left\{2R_{1}^{2}\sigma_{1}-\left(\frac{3}{2}R_{1}^{2}+\frac{1}{2}R_{2}^{2}\right)\sigma_{2}\right\}-\frac{1}{2E}\sigma_{\zeta }=0\;.\;$$

Therefore, it is possible to express the relationship between the contact stresses $\sigma_{1}$ and $\sigma_{2}$ and the axial stress $\sigma_{\zeta }$:

(10) $$\sigma_{1}=\frac{R_{1}^{2}-R_{2}^{2}}{R_{1}^{2}\left(1+2\varepsilon \right)-R_{2}^{2}\left(1-\frac{2}{3}\varepsilon \right)}\sigma_{\zeta },\;\;\sigma_{2}=\frac{\left(R_{1}^{2}-R_{2}^{2}\right)\left(1-\frac{2}{3}\varepsilon \right)}{R_{1}^{2}\left(1+2\varepsilon \right)-R_{2}^{2}\left(1-\frac{2}{3}\varepsilon \right)}\sigma_{\zeta }\;,$$

where $\varepsilon =\frac{ER_{1}}{E_{e}h}=18\frac{ER_{1}^{3}}{E_{0}h^{3}}\;.$

Substituting Equations (8) and (10) into relation (1), we obtain the key differential equation for axial stress:

(11) $$\frac{d\sigma_{\zeta }}{d\zeta }-\lambda \sigma_{\zeta }\mathrm{sgn}\zeta =0,\;\zeta \in \left(-1,1\right),$$

where the axial stress damping parameter is given by the following expression:

(12) $$\lambda =2a\frac{f_{1}R_{1}+f_{2}R_{2}\left(1-\frac{2}{3}\varepsilon \right)}{R_{1}^{2}\left(1+2\varepsilon \right)-R_{2}^{2}\left(1-\frac{2}{3}\varepsilon \right)}.$$

The boundary conditions at the ends of the hollow cylinder have the form:

(13) $$\sigma_{\zeta }\left(\pm 1\right)=-p\equiv -\frac{Q}{\pi \left(R_{1}^{2}-R_{2}^{2}\right)}.$$

## 3. Results and Analysis

The solution of Equation (11), under the boundary conditions of Equation (13), is given by the formula:

(14) $$\sigma_{\zeta }\left(\zeta \right)=-pe^{-\lambda (1-|\zeta |)}\;,\;\zeta \in \left(-1,\;\;1\right)\;.$$

According to the main result of Equation (14), on the basis of the relations of Equations (2), (3), (8), and (10), we can find all the characteristics of the stress–strain state of the system.

In particular, we obtain formulas for contact stresses:

$$\sigma_{1}(\zeta )=-p\frac{R_{1}^{2}-R_{2}^{2}}{R_{1}^{2}\left(1+2\varepsilon \right)-R_{2}^{2}\left(1-\frac{2}{3}\varepsilon \right)}e^{-\lambda (1-|\zeta |)},$$

$$\tau_{1}(\zeta )=f_{1}p\frac{R_{1}^{2}-R_{2}^{2}}{R_{1}^{2}\left(1+2\varepsilon \right)-R_{2}^{2}\left(1-\frac{2}{3}\varepsilon \right)}e^{-\lambda (1-|\zeta |)}\mathrm{sgn}\zeta ,\;$$

$$\sigma_{2}(\zeta )=-p\frac{\left(R_{1}^{2}-R_{2}^{2}\right)\left(1-\frac{2}{3}\varepsilon \right)}{R_{1}^{2}\left(1+2\varepsilon \right)-R_{2}^{2}\left(1-\frac{2}{3}\varepsilon \right)}e^{-\lambda (1-|\zeta |)},$$

(15) $$\tau_{2}(\zeta )=-f_{2}p\frac{\left(R_{1}^{2}-R_{2}^{2}\right)\left(1-\frac{2}{3}\varepsilon \right)}{R_{1}^{2}\left(1+2\varepsilon \right)-R_{2}^{2}\left(1-\frac{2}{3}\varepsilon \right)}e^{-\lambda (1-|\zeta |)}\mathrm{sgn}\zeta \;;\;$$

and for a push rod draft expression:

(16) $$\delta =\mp u\left(\pm 1\right)=\frac{pa}{E}\frac{2\varepsilon R_{1}^{2}}{R_{1}^{2}\left(1+2\varepsilon \right)-R_{2}^{2}\left(1-\frac{2}{3}\varepsilon \right)}\frac{1-e^{-\lambda }}{\lambda }.\;$$

The maximum stresses in the orthotropic shell is compared with the equivalent allowable stress [98]

$$\underset{\zeta }{\max}\sigma_{\vartheta 1}^{sh}(\zeta )=-\sigma_{1}(\pm 1)\frac{R_{1}}{h}=\frac{QR_{1}}{\pi h\left(R_{1}^{2}\left(1+2\varepsilon \right)-R_{2}^{2}\left(1-\frac{2}{3}\varepsilon \right)\right)}\leq {\left[\sigma \right]}_{e}\equiv \frac{h}{R_{1}}\frac{{\left[\sigma \right]}_{0}}{12}\;,$$

where ${\left[\sigma \right]}_{0}$ is an allowable stress for the shell material. We obtain the formula for the maximum operating load:

(17) $$Q_{\max}\leq \frac{\pi h^{2}}{12}{\left[\sigma \right]}_{0}\left\{1+2\varepsilon -\frac{R_{2}^{2}}{R_{1}^{2}}\left(1-\frac{2}{3}\varepsilon \right)\right\}\;.$$

Let us sum up the results obtained. As follows from Equation (15), the contact pressure on the shaft is always less than that on the outer shell. This effect is so noticeable that, for compliant shells (capital ε), the phenomenon of the detachment of the filler from the inner shell may occur. To prevent this from happening—that is, to ensure the condition $\sigma_{2}<0$—inequality $1-\frac{2}{3}\varepsilon >0\;,$ or ε<1.5  must be met.

The impact of the shaft also manifests itself in the fact that the presence of friction on the inner surface of the contact leads to a significant increase in the parameter λ and an exponential decrease in the axial and contact stresses (see Equation (12)). This effect gets stronger as the radius $R_{2}$ increases. As can be seen from Equations (16) and (17), the presence of an inner shell also leads to a slight increase in the compliance of the elastic element and a decrease in its holding capacity.

To illustrate the obtained analytical results, let us choose as an example the one shown in Figure 2—a system with parameters within the range of the operating characteristics of real shell damper designs $a/R_{1}=3$, $h/R_{1}=0.15$, $R_{1}/R_{2}=4$, $f_{1}=f_{2}=0.5$, and $E/E_{0}=0.0001$. The specified values will be used by default, however, in the process of analyzing the behavior of the damper. We will vary some of them, which will be indicated in the description of each specific graph. All calculations and plotting of the graphical dependencies were carried out using Mathcad, a computer algebra system from the class of computer-aided design systems.

**Figure 3:**
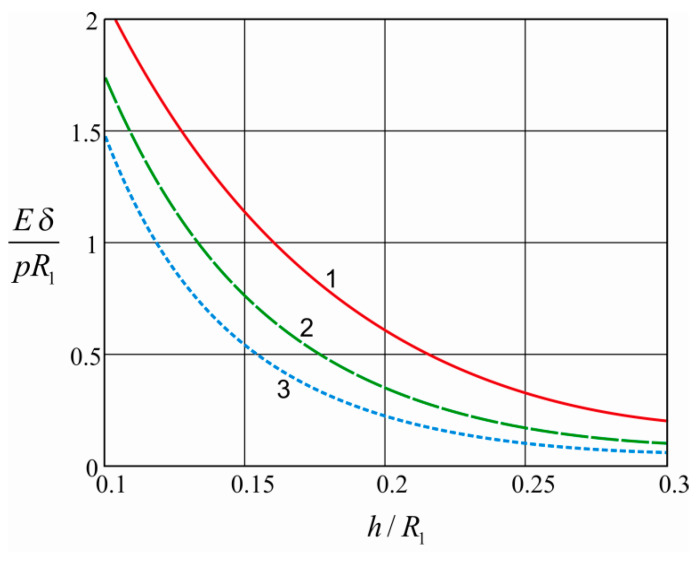
Influence of open shell thickness on damper compliance: 1—$f_{1}=f_{2}=0.2$; 2—$f_{1}=f_{2}=0.5$; 3—$f_{1}=f_{2}=0.8$.

Figure 3 graphically shows the dependences of the damper compliance on the thickness of the open shell for various friction coefficients of the shell-filler and barrel-filler contact pairs. We see that, as the shell thickness increases, the compliance of the system decreases. It should be noted that the dependence is non-linear and that the rate of this process gradually slows down. In other identical conditions, a system with a lower coefficient of friction in the contact pairs will have a higher compliance. An increase in the coefficient of friction leads to a decrease in the compliance of the elastic element.

**Figure 4:**
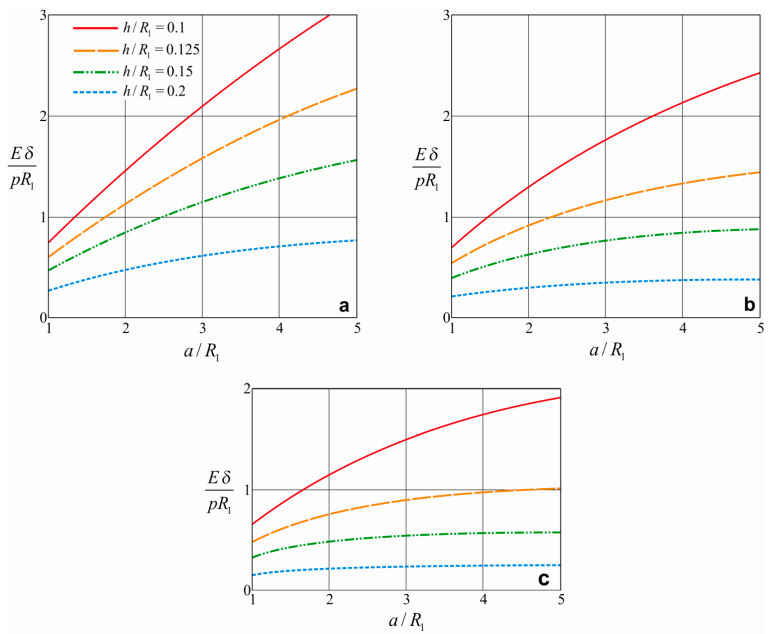
Effect of the relative length of the filler on damper compliance: (**a**) $f_{1}=f_{2}=0.2$; (**b**) $f_{1}=f_{2}=0.5$; (**c**) $f_{1}=f_{2}=0.8$.

Figure 4 shows the dependence of the compliance of the shell damper on the relative length of the filler for various shell thicknesses.

In general, with an increase in the length of the filler, the compliance of the damper increases, but this dependence is non-linear, and the following features can be traced. With an increase in the length of the system, the rate of the increase in compliance gradually decreases, especially when parameter λ becomes larger. This is because, due to friction, the contact pressure between the filler and the shell and between the filler and the shaft attenuates with distance from the ends of the filler. Thus, as the length of the damper increases, it turns out that the middle part of the shell works inefficiently. Therefore, an extensive increase in the length of the shell and filler is not a productive way to increase the compliance of the damper. The rate of the increase in compliance slows down more quickly in dampers with a larger open-shell wall thickness. Dependence analysis, according to Figure 4, allows us to talk about the optimal length of the system in each specific case [32,89].

**Figure 5:**
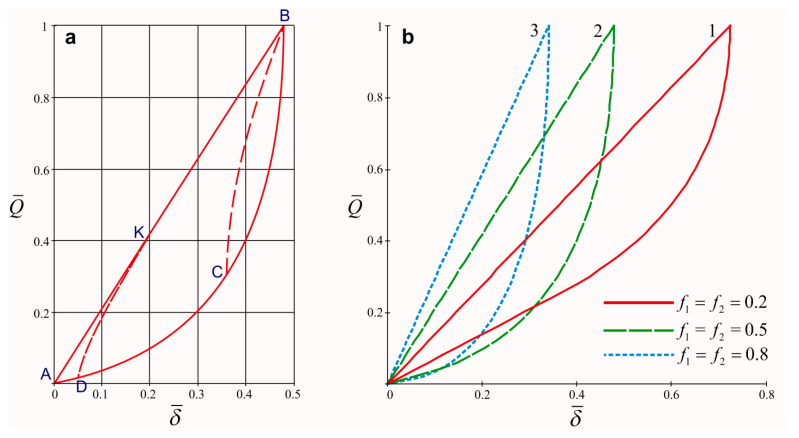
Diagram of the cyclic load of the shell damper: (**a**) solid lines: AB—active loading, BA—unloading; dashed lines CB тa DKB—repeated with different coefficients of cycle asymmetry; (**b**) the influence of the coefficient of friction of contact pairs on the deformation diagram: 1—$f_{1}=f_{2}=0.2$; 2—$f_{1}=f_{2}=0.5$; 3—$f_{1}=f_{2}=0.8$.

Using the method described in [30,31,90,91,93,99], we analytically investigated the quasi-static stress–strain state of the system in the case of nonmonotonic load Q behavior by time. The cyclic load diagram (structural damping loop) of the shell damper is shown in Figure 5a. Here, on the axes of the dimensionless coordinates $\bar{Q}=Q/Q_{\max}$, $\bar{\delta }=c\delta /Q_{\max}$, where $c=\frac{\pi \left(R_{1}^{2}-R_{2}^{2}\right)E}{a}\;\frac{1+2\varepsilon -\frac{R_{2}^{2}}{R_{1}^{2}}\left(1-\frac{2}{3}\varepsilon \right)}{2\varepsilon }\;$ is a linear rigidity of a conservative system. In the figure, the letters indicate the characteristic points of the diagram, allowing one to trace all of the stages of the load cycle. Solid lines show the diagram for a pulsating (zero) cycle: linear section AB—active loading; non-linear section BA—unloading. The dashed lines CB and DKB show the stages of repeated loading at different coefficients of cycle asymmetry $s=Q_{\min}/Q_{\max}$. The area bounded by the cyclic loading diagram is numerically equal to the amount of dissipated energy in one cycle.

Figure 5b illustrates the influence of a change in the value of the friction coefficient in the contact pairs of a shell damper on the form of the diagrams of cyclic deformation. The diagrams clearly show the effect of an increase in damper compliance with a decrease in the friction coefficient. At the same time, at a fixed amplitude of the soft load cycle, with an increase in the friction coefficient, the amount of energy dissipated per cycle gradually decreases (for example, the area of the damping loop at $f_{1}=f_{2}=0.8$ is visually smaller than that with $f_{1}=f_{2}=0.2$).

**Figure 6:**
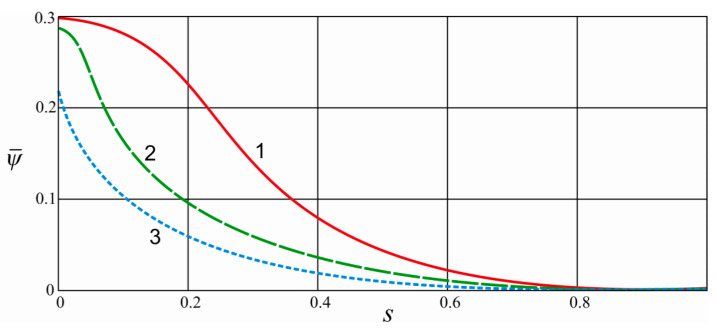
The dependence of the normalized value of the scattered energy on the cycle asymmetry coefficient: 1—$f_{1}=f_{2}=0.2$; 2—$f_{1}=f_{2}=0.5$; 3—$f_{1}=f_{2}=0.8$; coefficient of cycle asymmetry $s=Q_{\min}/Q_{\max}$.

Figure 6 shows the dependence of the normalized value of the scattered energy $\tilde{\psi }=\psi /A$ on the coefficient of asymmetry of the load cycle, where $A=Q_{\max}^{2}/\left(2c\right)$ is the energy of elastic deformation of a conservative system. With an increase in the cycle asymmetry coefficient, the amount of dissipated energy decreases, and the rate of such a decrease is higher for a damper with a large coefficient of friction in contact pairs.

The graphic dependences of Figure 3, Figure 4, Figure 5 and Figure 6, supplemented by Equation (17), allow one to vary the main operational parameters of the damper at the design stage, which, in turn, provides an opportunity to ensure the necessary efficiency of the vibration protection system as a whole.

## 4. Discussion

Globally, the idea of using the bending strain of load-bearing thin-walled links for shell dampers and shock absorbers is quite fruitful and will form the basis of a number of technical solutions. It should be noted that the shell damper has both damping and shock-absorbing properties. However, for the qualitative implementation of the theoretical concepts on which the principle of the operation of the damper is based, its constituent elements must have certain characteristic properties. The main energy storage (open shell) requires a combination of low rigidity with a sufficient level of strength and durability. For the directional transformation of displacements, the filler must easily change its shape (i.e., have a low shear modulus). From this point of view, the filler material should be soft compared to the shell material. On the other hand, in order to cause the open shell to deform under contact pressure, the filler material must have a high bulk modulus. These requirements are met by elastomers, which are also capable of frequency-dependent energy dissipation. The tribological characteristics of the filler-shell and filler-shaft contact pairs are selected in order to ensure the level of structural damping required in a particular operational situation.

As for the evaluation of the strength of the shell damper, for practical purposes, we have obtained Equation (17). Let us note that the maximum absolute value of the stress in the shell is the tensile hoop stress in the sections belonging to the planes of the ends of the filler. When monitoring the behavior of the contact stresses, it can be observed that they reach their maximum absolute values in the planes of the ends of the filler (at the edges of the contact zone), decreasing with distance from these planes.

The analytical construction of a diagram of the cyclic deformation of a shell damper can be considered a significant result of the study (Figure 5). With its help, using the known load history, it is possible to predict the behavior of the considered non-conservative system at any time after the start of the loading process, as well as to calculate the amount of energy dissipated in this case. Here, it is necessary to focus on some features of the damper deformation diagram. During the operation of the damper, structural energy dissipation is present only in the areas of the mutual slippage of the filler and the shell as well as the filler and the shaft. The movement of the piston at the stage of active loading is a linear function of Q—section AB (Figure 5a). This means that, at this stage, the adhesion in the contact pairs is not achieved. At the beginning of the unloading stage (nonlinear section BA), the conditions of non-unilateral slippage are satisfied on the contact surfaces. In other words, the contact surfaces are divided into areas in which the rate of the mutual displacement of the contacting bodies and, consequently, the tangential contact stresses have opposite signs. Toward the end of the unloading stage, one-sided slippage is achieved on the contact surfaces, so the BA unloading section always ends in a straight line.

When the damper is reloaded, two different variants of its behavior are possible depending on the cycle asymmetry coefficient. The DKB section visualizes a variant in which the conditions of non-unilateral slippage (dashed curve DK) are met at the beginning of the repeated loading phase, and then the conditions of one-sided slippage are met, and the diagram goes to the straight line of active loading (KB line). The CB section visualizes a reloading option in which one-sided slippage on the contact surfaces is never achieved. In this case, a number of areas with opposite signs of tangential contact stresses may appear on the contact surfaces. There can be any number of such areas with an arbitrary number of loading cycles and an arbitrary amplitude, and it depends solely on the loading history.

**Figure 7:**
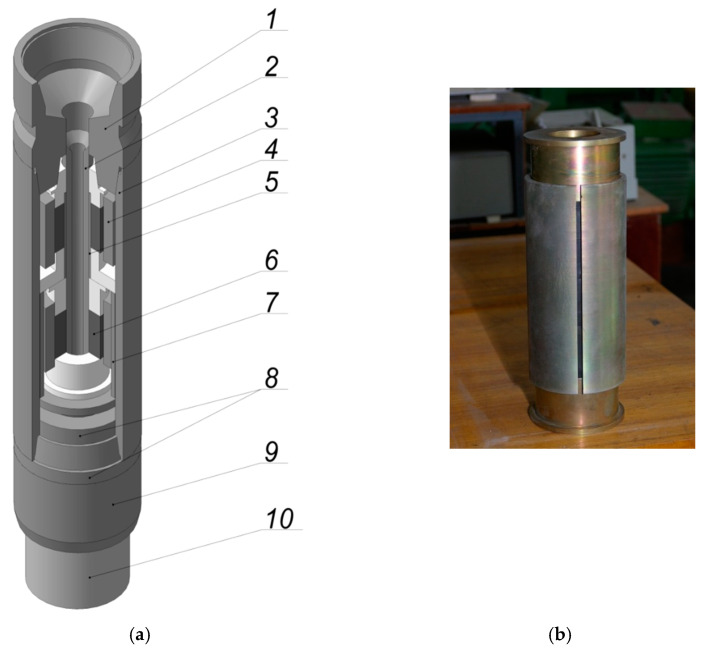
Schematic diagram of the drilling shock absorber (**a**), full-scale view of the shell damper (**b**): 1—shock absorber adapter, 2—shaft, 3—body, 4—first-stage damper, 5—support bushing, 6—second-stage damper, 7—first-stage damper engagement bushing, 8—profile pair of torque transmission, 9—centralizer, 10—bit adapter.

Dry friction dampers are quite often used in vibration protection systems, primarily due to the simplicity and manufacturability of the designs and the low-cost maintenance. The proposed open-shell damper is predicted to be successfully used in the mining and oil and gas industries. In particular, in oil and gas drilling techniques, a drill string is a long structure connecting downhole tools and a drilling rig. Its dynamic characteristics are one of the key factors affecting the efficiency and cost of drilling. Figure 7a shows a schematic diagram of a drilling shock absorber, which is designed on the basis of a dry friction shell damper. The main bearing link of this shock absorber is an open cylindrical shell (Figure 7b). The peculiarity of the presented model is the parallel operation of the elastic links of the shock absorber, which allows one to adjust the operational characteristics of the shock absorber at the design stage. Due to the presence of bushings 5 and 7, dampers 4 and 6 are integrated into parallel operation, while each damper bears a part of the external load, which is proportional to its own rigidity. It should be noted that, if necessary, it is possible to install a larger number of links, which will gradually (with an increase in the external load) be integrated into parallel operation.

We conclude with a few words about the reliability of the analytical results obtained in this article. The key point in our simulation is the replacement of an open shell working in bending with an equivalent continuous shell working in tension. It is assumed that both shells provide the same increase in the internal volume under pressure. This idea has already been used by the authors for the analytical modeling of the deformation of a cut cylindrical shell with incompressible filler in the absence of an internal shaft [98]. The validity of this approach and the accuracy of the results sufficient for engineering practice are confirmed by FEM simulation [19,23] and experimental data [101]. Moreover, in the limiting case $R_{2}\to 0$ from the results obtained in the third section, we obtain the corresponding results for a cut damper without a shaft [98]. All of the above is an argument in favor of the adequacy of our study.

## 5. Conclusions

The original design of a shell-type dry friction damper is presented. Its components are an open shell, elastomeric filler, a chrome-plated shaft, and rigid annular pistons. When well designed, such dampers combine a high holding capacity with relatively low rigidity and the required level of vibration damping. The use of an open-shell damper in heavily loaded systems with established restrictions on the transverse dimension of vibration protection devices and the possibility of the presence of aggressive and abrasive-containing media is predicted.

A technique for designing the presented damper has been developed. At the stage of setting the problem, a number of substantiated assumptions were introduced, which made it possible to effectively conduct research in an analytical way. Adequate in terms of rigor, models of shell damper components have been developed. The problem of the contact interaction of elastomeric filler with a coaxially installed cut cylindrical shell and a shaft is formulated and solved. Analytical expressions are obtained for estimating the strength and rigidity of the damper.

With a nonmonotonic load of the damper under consideration, due to the frictional interaction of the filler with the shell and with the barrel, part of the energy that is supplied to the system will be dissipated. The phenomenon of structural hysteresis arises in a non-mobile deforming system, which we studied in a quasi-static formulation. The end result of this stage of the study is a piecewise monotonic load diagram of the damper, taking into account all the essential parameters of the contact problem. Using the obtained results, we studied the dependence of the amount of dissipated energy on the coefficient of asymmetry of the external loading cycle.

The question of the dynamics of a specific mechanical system with an element of the hysteresis type has not yet been studied and will be the subject of future research by the authors.

The authors see good prospects for using the presented friction shock absorber in the construction, mining, and oil and gas industries.

## Data Availability

Data are contained within the article.
